# Inverted U-shaped response of a standardized extract of *Centella asiatica* (ECa 233) on memory enhancement

**DOI:** 10.1038/s41598-019-44867-z

**Published:** 2019-06-10

**Authors:** Yingrak Boondam, Phanit Songvut, Mayuree H. Tantisira, Sompol Tapechum, Kanokwan Tilokskulchai, Narawut Pakaprot

**Affiliations:** 1 Department of Physiology, Faculty of Medicine Siriraj Hospital, Mahidol University, Bangkok, Thailand; 2 Department of Pharmacology and Physiology, Faculty of Pharmaceutical Sciences, Chulalongkorn University, Bangkok, Thailand; 3 Faculty of Pharmaceutical Sciences, Burapha University, Chonburi, Thailand

**Keywords:** Extracellular recording, Pharmaceutics, Hippocampus, Spatial memory, Alzheimer's disease

## Abstract

*The herb Centella asiatica* has long been considered a memory tonic. A recent review found no strong evidence for improvement of cognitive function, suggesting negative results were due to limitations in dose, standardization and product variation. We used a standardized extract of *C. asiatica* (ECa 233) to study behavioral, cellular and molecular effects on learning and memory enhancement. ECa 233 (10, 30, and 100 mg/kg) was given orally to normal rats twice a day for 30 days. We used the Morris water maze to test spatial learning and performed acute brain slice recording to measure changes of synaptic plasticity in the hippocampus, a core brain region for memory formation. Plasticity-related protein expressions (NR2A, NR2B, PSD-95, BDNF and TrkB) in hippocampus was also measured. Rats receiving 10 and 30 mg/kg doses showed significantly enhanced memory retention, and hippocampal long-term potentiation; however, only the 30 mg/kg dose showed increased plasticity-related proteins. There was an inverted U-shaped response of ECa 233 on memory enhancement; 30 mg/kg maximally enhanced memory retention with an increase of synaptic plasticity and plasticity-related proteins in hippocampus. Our data clearly support the beneficial effect on memory retention of a standardized extract of *Centella asiatica* within a specific therapeutic range.

## Introduction

Aging population is a global problem, affecting both patient’s and caregiver’s health, while patient mental decline is a major burden in caregiving. Though the world is moving forward with new innovations, many diseases are not cured. Traditional herbal plants used in Ayurvedic medicine are promoted as candidates for health treatment or supplementary nutrition. *Centella asiatica* (L.) Urban (Indian pennywort, Gotu kola or Bua-bok) is a popular herbal plant used for more than a thousand years. It promotes longevity and wound healing, and shows antidepressant and neuroprotective properties^1^. The major bioactive constituents of *C. asiatica* are the triterpenoid glycosides including asiaticoside and madecassoside. They are contained in very low amounts that vary greatly in each plant. Although many studies have investigated the effect of *C. asiatica* extract on disease models with promising results, there are still conflicting data regarding the effect of *C. asiatica* on cognitive functions, as reviewed by Puttarak (2017)^2^. One possible reason for conflicting data might be different, unstandardized, plant extracts used in each experiment, with variation in each lot due to cultivation method, climate, etc. To overcome these problems, a standardized extract of *C. asiatica* (ECa 233) was established. ECa 233 is a white to off-white powder with triterpenoid glycosides not less than 80% w/w and the ratio of madecassoside and asiaticoside is maintained at 1.5 ± 0.5^3^.

ECa 233 has been tested for toxicology and pharmacokinetic activity. In acute toxicity testing, mice received ECa 233 orally up to 10 g/kg without lethality over an observation period of 14 days, whereas in chronic toxicity tests, rats received ECa 233 orally up to 1 g/kg for 90 days without any toxic effects^4^. ECa 233 also did not change CYP450, drug-metabolizing enzymes (DMEs) in phase I^5^ –, and DMEs in phase II of drug metabolism such as UDPGT, GST, SULT and NQOR^6^. Distribution of both asiaticoside and madecassoside are found in skin, stomach, and brain, which can be detected 1 hr after oral and intravenous administrations of ECa 233^3^.

Neuropharmacological activities of ECa 233 have been reported in many previous studies. For example, ECa 233 showed an anxiolytic effect in stress-induced rats, who demonstrated better performance in behavioral tasks than untreated rats^7^. ECa 233 also showed a neuritogenic effect, promoting neurite outgrowth on neuroblastoma cells via ERK1/2 and Akt pathways^8^. Moreover, ECa 233 demonstrated neuroprotective effects by attenuating the learning and memory deficit induced by transient bilateral occlusion of common carotid arteries (T2VO)^9^ or cerebral infusion of amyloid beta peptide 25–35 fragments (Aβ_25–35_)^10^. These neuroprotective effects were proposed to act through anti-oxidant and anti-inflammatory mechanisms of ECa 233.

Although previous studies have demonstrated the neuroprotective effects of ECa 233, the underlying mechanisms have not been fully demonstrated, and very few have investigated the memory enhancing effect of the compound in a normal condition. This is of utmost importance in understanding the real effect of the compound before and after the occurrence of brain insults. Synaptic plasticity is a cellular characteristic of memory formation, generated by the activation of N-methyl-D-aspartate receptor (NMDAR) following glutamate binding, especially in the CA1 region of the hippocampus. The triggering of NMDA signaling cascade mediates the activation of PKA, CREB and the transcription of brain neurotrophic factor (BDNF), respectively^11^. BDNF promotes the growth of original synapses and formation of new synapses through binding with its receptor, tyrosine kinase B (TrkB). This is the basis of synaptic plasticity in learning and memory formation^12^. Therefore, we focused on the effects of a standardized extract of *C. asiatica* (ECa 233) on learning and memory in the normal rat model, with a goal of demonstrating effects and underlying mechanisms. Enhanced memory performance was measured by results of the Morris water maze task, and synaptic enhancement in the hippocampus was assessed by measuring long-term potentiation magnitude, while expression of plasticity-related proteins following NMDAR-signaling activation was examined by western blot analysis. We also confirmed distribution of major bioactive constituents in the hippocampus and plasma using the liquid chromatography tandem-mass spectrometry (LC-MS/MS) technique.

## Results

### Quantification of madecassoside and asiaticoside following ECa 233 treatment

Since the major bioactive constituents of ECa 233 are madecassoside and asiaticoside, quantification of these two constituents is required to confirm the distribution of substances in both plasma and hippocampus, the critical brain region for spatial memory formation. After treatment with ECa 233 for 30 days, results showed the presence of both madecassoside and asiaticoside in plasma (Table 1) [*F*(3,19) = 23.637, *P* < 0.001] and [*F*(3,19) = 543.539, *P* < 0.001], and in the hippocampus (Table 2) [*F*(3,19) = 205.715, *P* < 0.001] and [*F*(3,19) = 166.170, *P* < 0.001] in a dose-dependent manner. Distribution of madecassoside and asiaticoside in the hippocampus were in accord with the ratio of these two constituents in ECa 233. The presence of active ingredients measured in the plasma and hippocampus confirms the presence of ECa 233 in systemic circulation and also in the brain, which may contribute to the memory enhancing effect of ECa 233.

**Table 1:** Quantification of madecassoside and asiaticoside in plasma using LC-MS/MS technique.

Dose (ECa233)	Mean concentration in plasma (ng/ml)	
	Madecassoside	Asiaticoside
0 mg/kg (control)	<LLOQ	<LLOQ
10 mg/kg	3.55 ± 0.24^a,d^	<LLOQ
30 mg/kg	5.42 ± 0.73^a,d^	<LLOQ
100 mg/kg	10.11 ± 1.55^a,b,c^	2.74 ± 0.11^a,b,c^

LLOQ = Lower limit of quantification.

Data are shown as mean ± SEM of 5 animals.

^a^*P* < 0.05 compared to the control group.

^b^*P* < 0.05 compared to the 10 mg/kg ECa-treated group.

^c^*P* < 0.05 compared to the 30 mg/kg ECa-treated group.

^d^*P* < 0.05 compared to the 100 mg/kg ECa-treated group.

**Table 2:** Quantification of madecassoside and asiaticoside in the hippocampus using LC-MS/MS technique.

Dose (ECa233)	Mean concentration in brain (ng/g brain)	
	Madecassoside	Asiaticoside
0 mg/kg (control)	<LLOQ	<LLOQ
10 mg/kg	151.19 ± 3.30^a,c,d^	106.97 ± 65.83^a,c,d^
30 mg/kg	555.45 ± 96.89^a,b,d^	425.57 ± 81.25^a,b,d^
100 mg/kg	1006.25 ± 75.44^a,b,c^	770.17 ± 71.61^a,b,c^

LLOQ = Lower limit of quantification.

Data are shown as mean ± SEM of 5 animals.

^a^*P* < 0.05 compared to the control group.

^b^*P* < 0.05 compared to the 10 mg/kg ECa-treated group.

^c^*P* < 0.05 compared to the 30 mg/kg ECa-treated group.

^d^*P* < 0.05 compared to the 100 mg/kg ECa-treated group.

### Enhancing effect of ECa 233 on spatial memory performance

**Figure 1:**
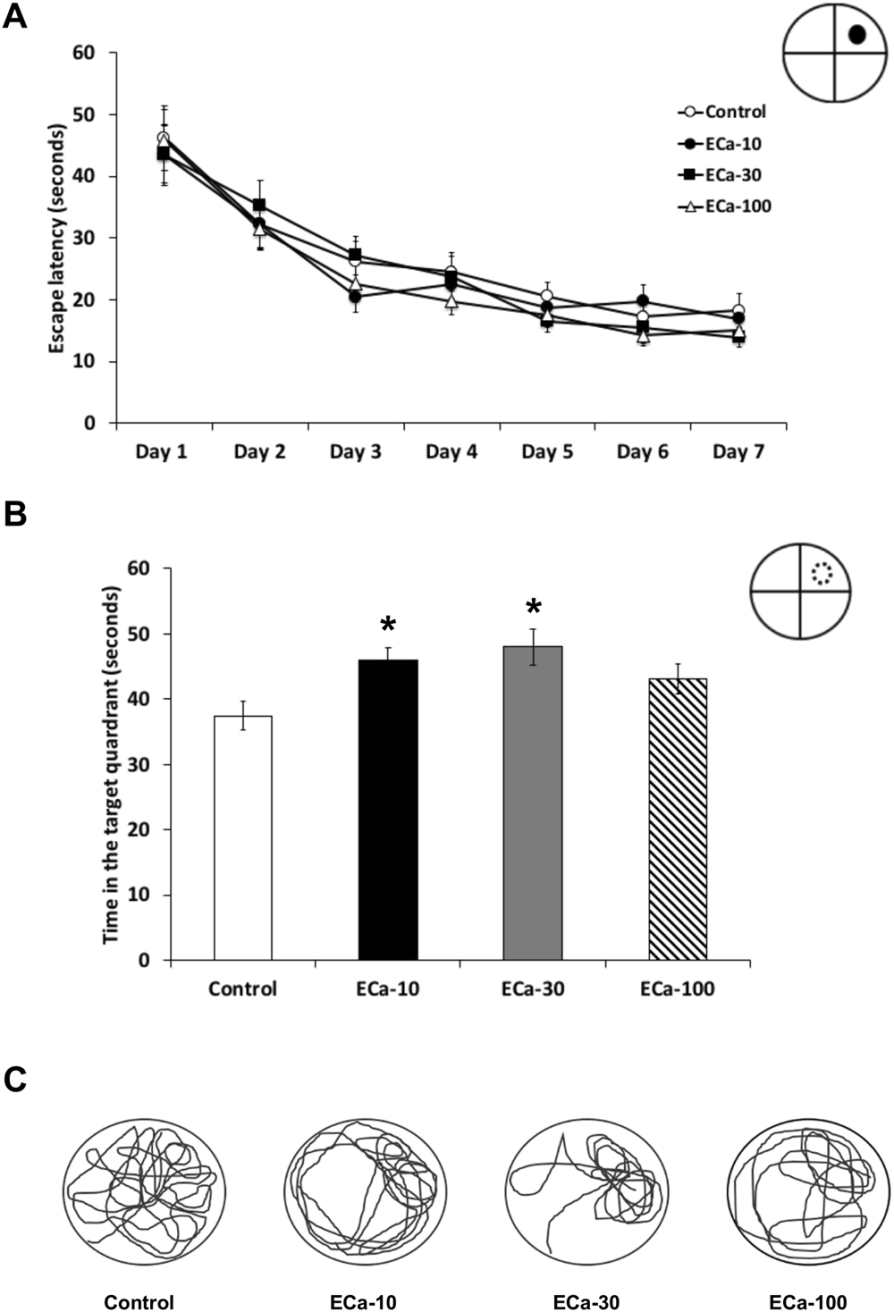
The effect of ECa 233 on spatial learning and memory. (**A**) The escape latency during seven consecutive days of the acquisition trial. (**B**) The time spent in the target quadrant during the probe trial. (**C**) A representative sample of swim traces on the probe trial. The behavioral test was performed 22 days after substance administration. Values are expressed as mean ± SEM of 14 animals. *P < 0.05 compared to the control group.

The enhancing effect of ECa 233 on spatial memory was tested by the Morris water maze task, where rats are challenged to swim to a formerly visible, now hidden platform. All rats treated with ECa 233 in all dosages showed no significant difference in the latency to find the platform (escape latency) over seven days of training (Fig. 1A). The statistical data at the 7^th^ day showed as follows *F*(2.489,32.357) = 0.674, *P* = 0.548. However, rats receiving ECa 233 at the doses of 10 and 30 mg/kg significantly increased the time spent in the targeted quadrant (Fig. 1B,C) [*F*(3,55) = 3.962, *P* = 0.013] compared to the control group. These results suggest that ECa 233 at the doses of 10 and 30 mg/kg might significantly promote memory retention.

### Enhancing effect of ECa 233 on hippocampal long-term potentiation

**Figure 2:**
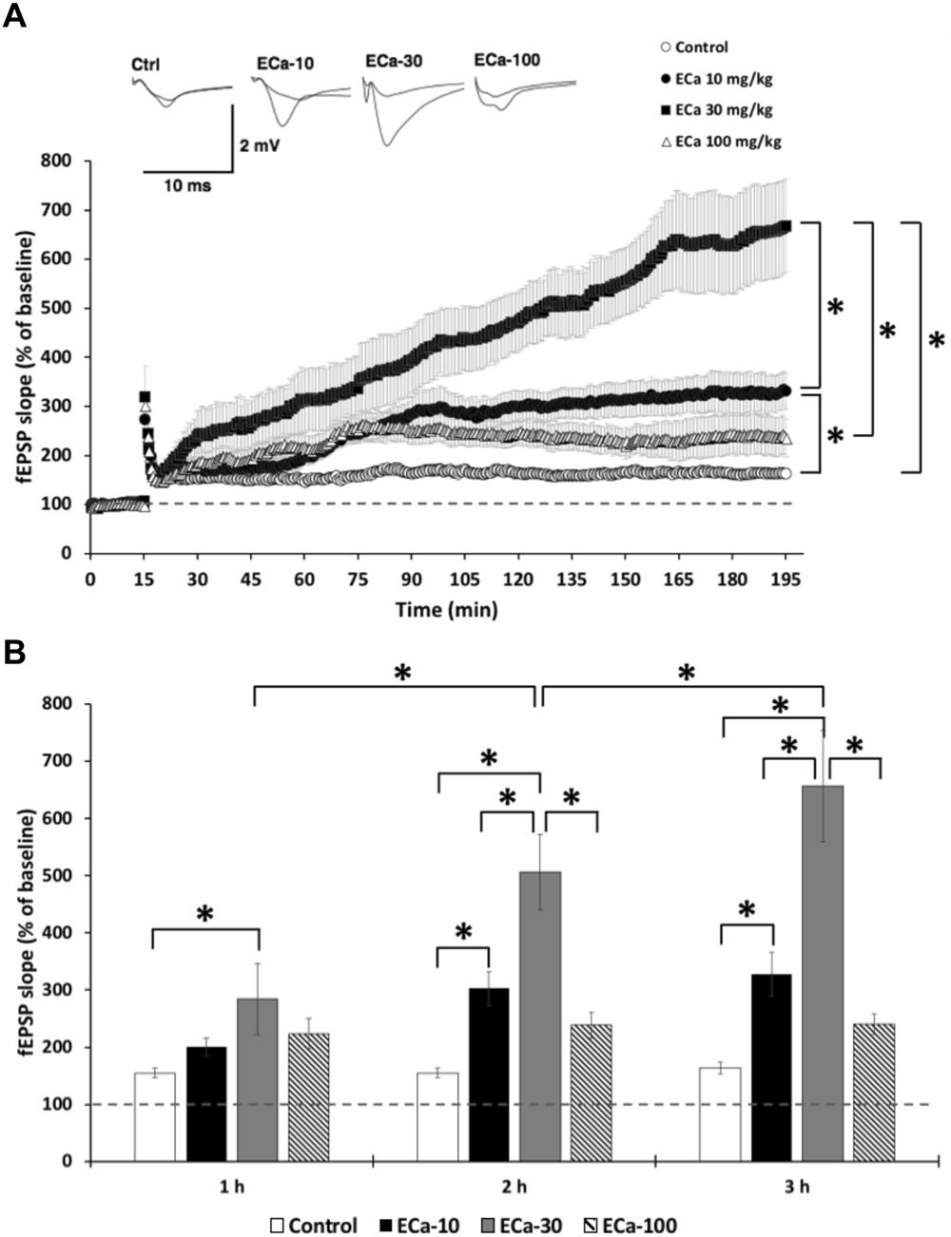
The effect of ECa 233 on long-term potentiation (LTP) magnitude. (**A**) The enhancement of LTP magnitude throughout 3 hr after high-frequency stimulation (HFS). (**B**) The averaged percentage of baseline fEPSP slope of the last ten minutes at each time point. The dotted line represents the percentage of baseline before HFS. Values are expressed as mean ± SEM of 7 animals. *P < 0.05 compared between groups.

The enhancing effect of ECa 233 on synaptic plasticity was demonstrated by the presence of hippocampal LTP enhancement, which is a characteristic of synaptic plasticity during memory formation. We found that ECa 233 at the dose of 10 and 30 mg/kg could increase hippocampal synaptic plasticity whereas ECa 233 at the dose of 100 mg/kg did not show any improvement. ECa 233 at the dose of 30 mg/kg gradually and significantly enhanced LTP magnitude from the 23^rd^ minute after HFS and until the end of recording (3 hr) when compared to the control group. However, at the dose of 10 mg/kg, ECa 233 began to significantly enhance synaptic plasticity 2 hr later after LTP induction when compared to the control group (Fig. 2A,B) [group effect: *F*(3,72) = 33.667, *P* = < 0.001; time effect: *F*(2,72) = 6.336, *P* = 0.003; group x time interaction: *F*(6,72) = 3.287, *P* = 0.007; *P* = 0.054 at 1^st^ hr, *P* < 0.001 at 2^nd^ hr, *P* < 0.001 at 3^rd^ hr].

### Effect of ECa 233 on the expression of plasticity-related proteins

**Figure 3:**
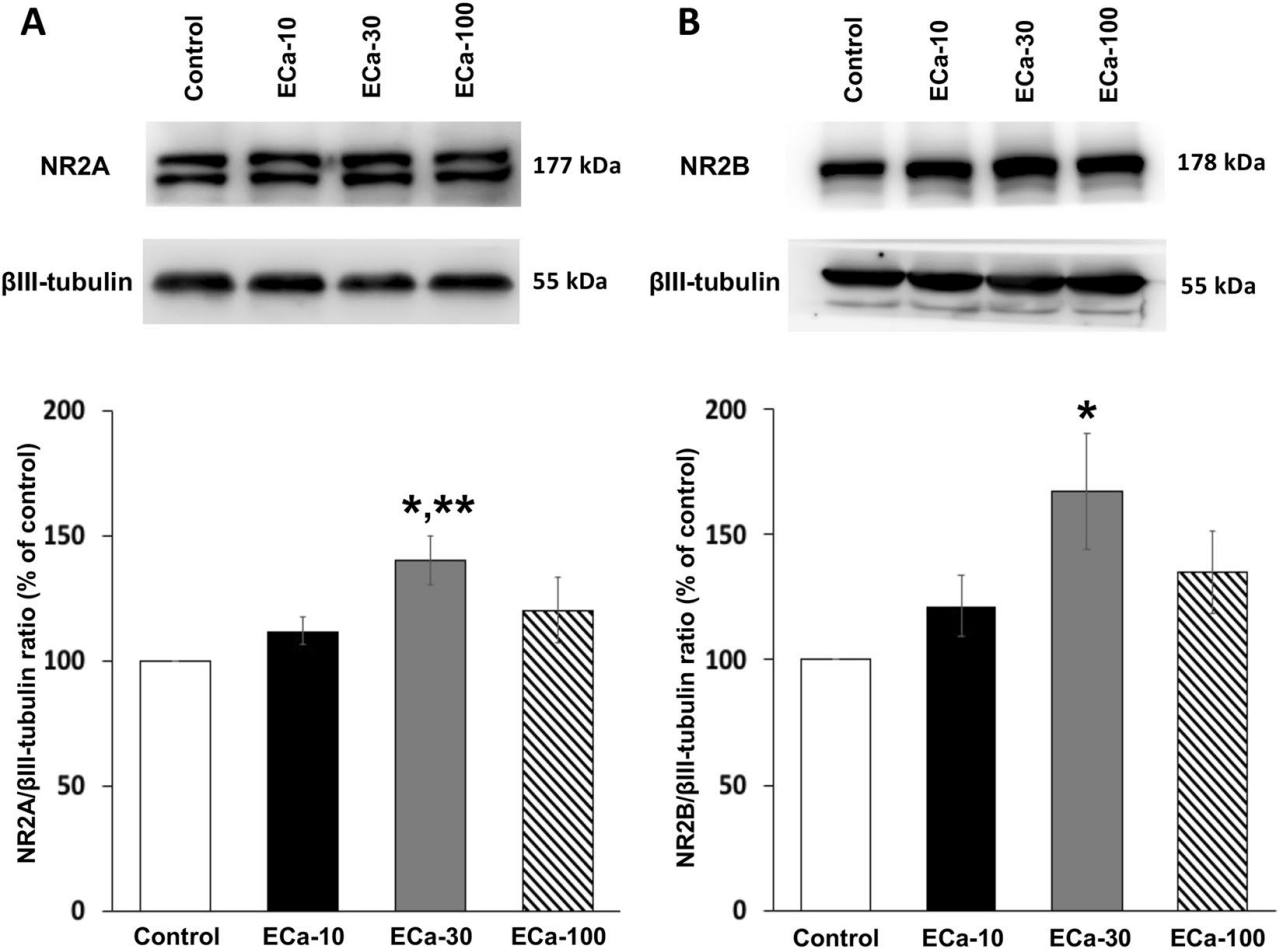
The effect of ECa 233 on NR2 subunit expression. (**A**) Representative western blotting for NR2A and relative levels of NR2A protein compared to total βIII-tubulin protein. (**B**) Representative western blotting for NR2B and relative levels of NR2B protein compared to total βIII-tubulin protein Values are expressed as mean ± SEM of 6 animals. *P < 0.05 compared to the control group and **P < 0.05 compared to the 10 mg/kg ECa 233-treated group. Full-length blot of NR2A and NR2B subunit protein is presented in Supplementary Figs S1 and S2, respectively.

**Figure 4:**
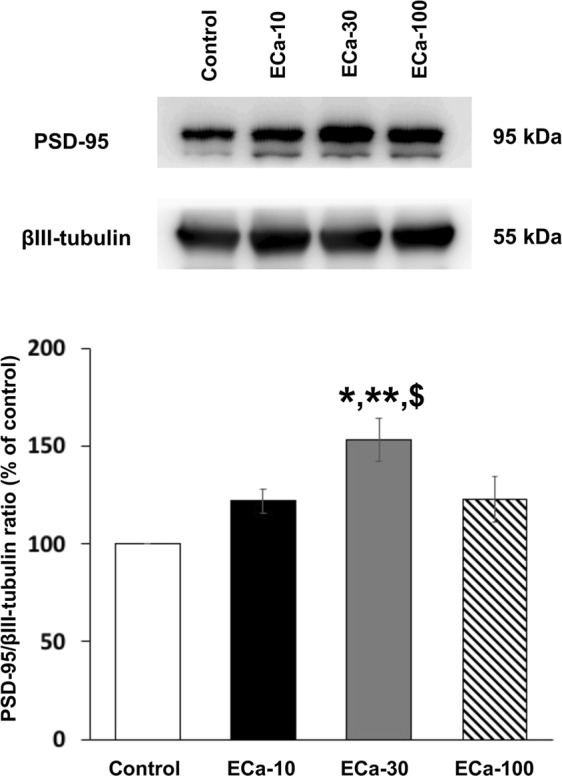
The effect of ECa 233 on a PSD-95 scaffolding protein expression. Representative western blotting for PSD-95 and relative levels of PSD-95 protein compared to total βIII-tubulin protein. Values are expressed as mean ± SEM of 6 animals. *P < 0.05 compared to the control group, **P < 0.05 compared to the 10 mg/kg ECa-treated group, and ^$^*P* < 0.05 compared to the 100 mg/kg ECa-treated group. Full-length blot is presented in Supplementary Fig. S3.

**Figure 5:**
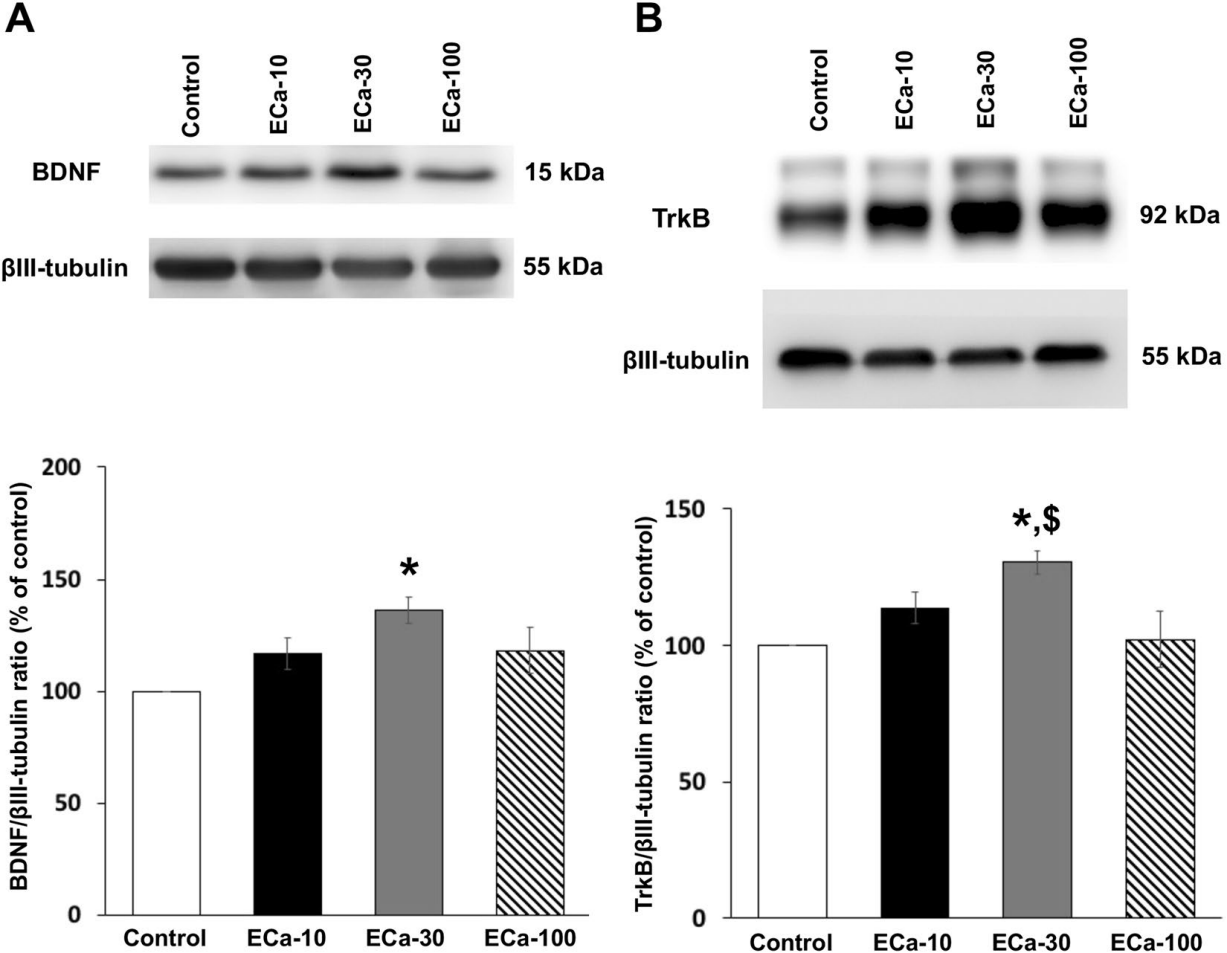
The effect of ECa 233 on BDNF and TrkB – BDNF receptor – proteins expression. (**A**) Representative western blotting for BDNF and relative levels of BDNF protein compared to total βIII-tubulin protein. (**B**) Representative western blotting for TrkB and relative levels of TrkB protein compared to total βIII-tubulin protein. Values are expressed as mean ± SEM of 6 animals. *P < 0.05 compared to the control group and ^$^*P* < 0.05 compared to the 100 mg/kg ECa-treated group. Full-length blot is presented in Supplementary Figs S4 and S5, respectively.

Synaptic plasticity in CA1 region of the hippocampus is associated with the activation of NMDAR signaling cascade. NR2A and NR2B are the major subunits of NMDAR, expressed in the rat hippocampus. ECa 233 at the dose of 30 mg/kg induced a dramatic increase in NR2A levels (Fig. 3A) [*F*(3,23) = 3.862, *P* = 0.030] and NR2B levels (Fig. 3B) [*F*(3,23) = 3.374, *P* = 0.045] compared to the control group. The levels of PSD-95, which is a scaffolding protein binding with both NR2 subunits, were significantly increased in ECa 233-treated rats at the dose of 30 mg/kg (Fig. 4) [*F*(3,23) = 6.433, *P* = 0.005] compared to the control group. The levels of BDNF, which is a group of neurotrophic factors promoting synaptic growth and formation, were significantly increased in ECa 233-treated rats at the dose of 30 mg/kg (Fig. 5A) [*F*(3,23) = 1.951, *P* = 1.154; followed by LSD post hoc, *P* = 0.025]. In addition, TrkB – a receptor of BNDF – was dramatically increased only at the dose of 30 mg/kg of ECa 233 (Fig. 5B) [*F*(3,23) = 4.894, *P* = 0.013]. Thus, ECa 233 at the dose of 30 mg/kg could enhance the expression of plasticity-related proteins, which is a downstream effect of NMDAR signaling cascades.

## Discussion

We investigated the memory enhancing effect of a standardized extract of *Centella asiatica* (ECa 233) on learning and memory in normal rats. Previous studies have shown that ECa 233 improved memory deficit in the T2VO- and Aβ-induced cognitive impairment models, but the underlying mechanisms of the memory improvement have not been fully elucidated. In this experiment, the Morris water maze task was used for assessment of spatial learning and memory performance, following ECa 233 treatment at the doses of 10, 30 and 100 mg/kg compared to the control group. We found that although ECa 233 in all dosages did not change the learning performance of the animals, ECa 233 at the doses of 10 and 30 mg/kg enhanced the retention of previously acquired memory. These behavioral findings were consistent with our electrophysiological and protein analysis results. In the electrophysiological study, the hippocampal synaptic responses (LTP magnitude) showed a gradual increase throughout 3 hr following the treatment of ECa 233 at the doses of 10 and 30 mg/kg, even though the onset showing significant enhancement of the 10 mg/kg dose occurred later than that of the 30 mg/kg dose. Moreover, in the protein analysis the expression of all plasticity-related proteins (including NR2A, NR2B, PSD-95, BDNF, and TrkB) were highly up-regulated at the dose of 30 mg/kg and tended to decrease at the dose of 100 mg/kg.

It is widely accepted that the hippocampus is an important brain region for spatial memory processes. Memory formation starts with the information encoding process followed by the rehearsal process. These two stages lead to short-term memory formation, and this short-term spatial memory is consolidated to long-term memory by the hippocampus^13,14^. In the Morris water maze task, during the acquisition trials, rats have to encode the visual cues and match the visual cues with the platform position. The rats must mentally rehearse the platform position by using the visual cues to navigate themselves to the platform for seven training days. Then, this acquired long-term memory is assessed by determining the time spent in the target quadrant during the probe trial^15^. Enhancement of memory retention by the tested rats was shown after they received ECa 233 at the doses of 10 and 30 mg/kg. Although not statically significant, the dose of 30 mg/kg tended to show a greater memory enhancing effect than the dose of 10 mg/kg. These behavioral data seem to correspond well with the enhancement of LTP magnitude in the electrophysiology study and with the increased expression of signaling proteins by western blot analysis. In the LTP study, only the doses of 10 and 30 mg/kg significantly enhanced LTP magnitude, but the dose of 30 mg/kg showed faster and higher enhancement. However, in the protein analysis, only the dose of 30 mg/kg showed increased expression of signaling proteins.

LTP is the most popular and generally used physiological model of synaptic plasticity during the learning and memory processes^16^. The formation of LTP in the CA1 area of the hippocampus is NMDA receptor-dependent. Generally there are two phases of LTP, the early phase, which forms and lasts around 30 min to 2 hr^17^, and the late phase, which forms and lasts at least 24 hr^14,17,18^. Here we showed that ECa 233 at the doses of 10 and 30 mg/kg, but not 100 mg/kg, was able to enhance the late-LTP, which depends on the new protein synthesis through the activation of AMPA and NMDA receptor-signaling cascade^13^.

Interestingly, the pattern of ECa 233-induced LTP is different from the regular pattern of LTP formation. After high frequency stimulation (HFS), the expression of fEPSP slopes in all groups dramatically increased, and then declined to around 150–160% of the baseline slope. In the control group, the LTP magnitude was maintained at this level until the end of the study (3 hr). In contrast, after declining to the levels of 150–160% of the baseline slope, the ECa 233-induced LTP, especially at the dose of 30 mg/kg, gradually and progressively increased throughout the recording time. The ECa 233-induced LTP seemed to reach a stable plateau toward the end of the third hour of testing. This ECa 233-induced LTP pattern is consistent with the BDNF-induced LTP studied in the perforant path and the Schaffer collateral pathway^19,20^.

In our study, BDNF and TrkB expressions were enhanced after the ECa 233 administration at the dose of 30 mg/kg. The activation of PI3K/Akt signaling following BDNF/TrkB activation also promotes TrkB anterograde transport in the axon and surface insertion^21^, perhaps resulting in the increased expression of TrkB we observed. The binding of BDNF/TrkB mediates neuronal survival, axonal growth, dendritic spine formation, synaptic formation, synaptic transmission, and eventually enhanced synaptic plasticity, respectively^12,22^.

BDNF can also up-regulate the expression of NR2A and NR2B (the regulatory subunits of NMDAR)^23^, and PSD-95 proteins at the surface membrane^24^. We found only the rats receiving ECa 233 at the dose of 30 mg/kg (the same dose that enhanced BDNF and TrkB expressions) showed the enhanced expressions of NR2A, NR2B and PSD-95, a scaffolding protein forming the bridge connecting NMDARs and downstream signaling molecules^25^. Therefore, in the present study, ECa 233 might promote the synthesis of BDNF proteins, in which they would be transported and stored at glutamatergic synapses, and could be secreted into the synaptic cleft upon the HFS^26^. The secreted BDNF proteins then bind with TrkB, mediating the increased expression of synaptic plasticity associated proteins, and new dendritic protein synthesis seen in LTP.

The enhancement of plasticity-related protein expressions were in agreement with the study of Lin and colleagues in 2014^27^. They found that madecassoside, one of the two major active constituents of ECa 233, enhanced learning and memory behavior and up-regulation of PSD-95, p-NMDAR1 (NR1 subunit), p-CaMKII, p-PKACβ, p-CREB and BDNF in the cognitive-impairment model^27^. Although the substance used in our study is different from their study, the amount of madecassoside in ECa 233 at the dose of 30 mg/kg (about 15.3 mg/kg of madecassoside), is in a range of the effective dose of madecassoside (10 and 20 mg/kg) in the Lin and colleague study. Moreover, ECa 233 was able to promote neurite outgrowth in the neuroblastoma cells^8^. This neuritogenic effect was a result of the activation of MEK/ERK and PI3K/Akt pathways^28,29^. According to the study of Yin and colleagues in 2015^30^, asiaticoside, which is the other major active constituent of ECa 233, also promoted the expression of p-Akt, GSK-3, synaptophysin (SYN) and PSD-95 in high glucose-treated neuroblastoma cells^30^. They suggested that asiaticoside could mediate the alteration of presynaptic terminals because it promoted an expression of SYN, a synaptic vesicle membrane protein related to the release of glutamate vesicles from presynaptic terminals, and also promoted downstream Trk-signaling cascades^30,31^.

Moreover, besides the effects on NMDAR and BDNF, the effects of Centella asiatica extract on α-amino-3-hydroxy-5-methyl-4-isoxazolepropionic acid (AMPA) receptors – another glutamate receptor important for LTP formation, synaptic plasticity, spatial learning and memory – were reported. The 14-day oral administration of the extract at the dose of 30 mg/kg, but not 300 mg/kg, could enhance the expression of GluA1 subunit of AMPA receptor (AMPAR) in CA1 and CA2 subregions of the hippocampus along with the better learning and memory performances in the water T-maze task^32^. Recently, the same group reported the effects of the extract in higher doses (100, 300, and 600 mg/kg). The enhanced spatial learning and memory retention in the Morris water maze were observed after the extract administration only at the dose of 300 mg/kg. The authors argued that the insignificant effect of the extract at the doses of 100 and 600 mg/kg might be because the Morris water maze task required more cognitive demand than the bidirectional T-maze task. Although, in this later experiment, all high doses of the extract enhanced expressions of AMPAR and NMDAR subunits, there were differential expressions of AMPAR and NMDAR subunits in different hippocampal subregions (CA1 and CA3) and entorhinal cortex^33^. All of these effects of the extract especially at the high doses might have to be further examined in more detail.

In agreement with these previous studies, ECa 233 might act on AMPAR, NMDAR and BDNF/TrkB signaling cascades. Although it was also reported that BDNF could increase the expression of AMPA receptors^34^, we cannot directly answer which event occurs first in this study because although BDNF can regulate the expression of AMPARs, NMDARs and PSD-95, BDNF itself is also secreted following the activation of AMPAR and NMDAR^23,24,35^. Although our data suggest earlier BDNF rather than AMPAR and NMDAR activations, this question has to also be further investigated.

The results from the behavioral, LTP, and plasticity-related protein expression studies following ECa 233 treatment presented an inverted U-shaped dose-response relationship. The doses of 10 and 30 mg/kg showed enhancement of memory retention and synaptic plasticity. Only the dose of 30 mg/kg showed enhanced expression of the plasticity-related proteins, whereas the higher dose (100 mg/kg) of ECa 233 showed no beneficial effects. One of the reasons could be that the plant products may contain other constituents, which at a high dose can exert some antagonistic or inhibitory effects perhaps on the hippocampal circuitry. A previous study found that an aqueous extract of *Centella asiatica* increased glutamic acid decarboxylase activity, which is an enzyme involved in GABA synthesis^36^. Moreover, asiatic acid, an active metabolite of asiaticoside, can interact with GABA_B_ receptor, inhibiting the synaptic transmission at the Schaffer collateral pathway^37^. Moreover, Yeo *et al*. (2018) suggested AMPA receptor desensitization due to a persistent increase of glutamate after the high dose of Centella asiatica extract treatment (300 mg/kg) which did not show the AMPAR activation and memory enhancement exhibited by the lower dose (30 mg/kg)^32^. All of these possibilities could perhaps inhibit the beneficial effects of the major constituents, which may explain why the highest dose of ECa 233 showed results similar to the control group.

On the other hand, the lowest dose in this study (10 mg/kg) also showed enhanced memory retention and LTP magnitude, although the increased LTP magnitude induced by 10 mg/kg of ECa 233 was not as much as that induced by 30 mg/kg of ECa 233. Moreover, ECa 233 administration at the dose of 10 mg/kg did not significantly up-regulate the expressions of NR2A, NR2B, PSD-95, BDNF, and TrkB proteins. It is possible that at low doses, ECa 233 may act on other mechanisms that are not related to NMDAR and BDNF/TrkB cascades, such as Ephrin-B signaling which promotes the stabilization of AMPARs at the surface membrane, causing the increment of synaptic strength at the CA1 region^38^. This mechanism could also effectively enhance memory retention, and moderately increase LTP magnitude. So, the stimulation of NMDA and BDNF/TrkB cascades may require a higher dose of ECa 233 such as 30 mg/kg, and the low doses of ECa 233 might activate other mechanisms, resulting in enhanced memory retention. Also, ECa 233 is comprised of many components in addition to the main components (89%) madecassoside and asiaticoside. Since both madecassoside and asiaticoside stimulate NMDAR and BDNF cascades, it may require at least moderate doses of madecassoside and asiaticoside to show an effect. And it is possible that the other unknown components might also enhance memory retention through other mechanisms.

Furthermore, in our study, the effects of ECa 233 were supported by the presence of its major active constituents – madecassoside and asiaticoside – in both plasma and hippocampus, the brain structure important for learning and memory. According to the pharmacokinetic study of ECa 233 in rats, the maximum plasma concentrations (C_max_) of madecassocide and asiaticoside were observed within 5–15 min after oral administrations, and the concentrations gradually decreased over time, whereas the two active constituents accumulated in the tissues^3^. In the brain, the area under the curve of madecassoside and asiaticoside levels from time 0–4 hr after the oral treatment (AUC_0–4_) were 84 and 20 ng × h/g after a single dosing (1 day) of ECa 233 (100 mg/kg/day), whereas the AUC_0–4_ of madecassoside and asiaticoside were 309 and 120 ng × h/g after a multiple dosing (7 days), respectively^39^. This tissue accumulation was clearly demonstrated in our experiment in which the concentrations of madecassoside and asiaticoside in the hippocampus were 1,006.25 and 770 ng/g after the 30-day oral administration of ECa 233 (100 mg/kg/day). Nevertheless, the multiple oral dosing oppositely affected the plasma levels of the constituents. The C_max_ of madecassoside (948 ng/ml) and asiaticoside (210 ng/ml) from the multiple oral dosing (7 days) were much lower than the C_max_ of madecassoside (5,713 ng/ml) and asiaticoside (1,282 ng/ml) from the single dosing (1 day) of ECa 233 (100 mg/kg/day)^39^. The effect of multiple dosing might be due to the activation of efflux transporters induced by the active constituents. The efflux transporters would pump the active ingredients back into the gastrointestinal tract, causing the decreased levels of the active constituents in plasma after the multiple oral dosing^39^. In our case, the plasma concentrations of madecassoside (10.11 ng/ml) and asiaticoside (2.74 ng/ml) were much lower than the C_max_ of both constituents after the multiple oral dosing (7 days). The reasons might be due to the prolonged administration of ECa 233 (30 days) and the delayed duration in which the blood samples were taken. In our experiment, the cardiac blood was collected around 30–35 min after the treatment during the intracardiac perfusion to preserve the tissues, so the plasma concentrations would be lower than the C_max_ of both constituents.

In summary, we have demonstrated the enhancement of hippocampal synaptic plasticity represented by LTP magnitude and that of plasticity-related protein expressions underlying the memory enhancing effect of ECa 233 in normal rats. Our study revealed that ECa 233, a standardized extract of the herb *Centella asiatica*, at the dose of 30 mg/kg had a memory enhancing effect at least through the activation of BDNF/TrkB function and NMDAR-signaling cascade. These findings provide basic knowledge of ECa 233 effects on memory formation, and support the development of ECa 233 as a memory-rescue drug or a dietary supplement. The inverted U-shaped dose-response relationship might indicate one of the factors responsible for conflicting data in the previous review^2^. It may suggest that an effective dose of *Centella asiatica* extract should be limited to moderate doses in order to achieve beneficial effects on spatial memory performance. The recommended dose of ECa 233 in this study is 30 mg/kg, which showed the beneficial effects on memory retention, LTP magnitude, and levels of synaptic plasticity related proteins. Therefore, further drug development and clinical studies should be performed.

## Materials and Methods

### Animals

Fifty-six healthy male Wistar rats, aged 7 weeks and weighing 180–210 grams, were used in this study. The animals were purchased from The National Laboratory Animal Center, Mahidol University. All rats were housed under conditions of controlled humidity, temperature and light (12 hr day/night cycle) with free access to water and murine chow. All animal experiments were performed in concordance with the guideline for animal care and use in scientific study, and received the approval from the animal ethics committee of Faculty of Medicine Siriraj Hospital, Mahidol University before the experimentation (approval number SI-ACUP 010/2560 on January 9, 2018).

### Chemicals

ECa 233 is an off-white extracted powder of *Centella asiatica*. The compounds of the ECa 233 extract were analyzed by using HPLC ESI-QTOF-MS/MS technique compared with analytical standards, and the quantitative analysis was performed with a triple quadrupole LC-MS/MS system^40^. Two major components corresponding to the two major peaks in the analysis were madecassoside and asiaticoside. In addition, the detected minor components were centellasaponins. However, the specific types of centellasaponins (A, C or D) could not be determined due to the lack of commercially available centellasaponins. Moreover, two other minor components were madecassic and asiatic acids. These triterpenic acids were accounted for less than 1.0% of ECa 233. The obtained ECa 233 extract contained 51% madecassoside and 38% asiaticoside (Siam Herbal Innovation Co., Ltd., Thailand, prepared by a patent-pending method). Asiaticoside (>98.5%) and madecassoside (>95.0%) were purchased from Sigma-Aldrich Corp. Glycyrrhetinic acid (an internal standard, >99.0%) was obtained from Wako Pure Chemical Industries, Ltd.

### Drug administration

ECa 233 was suspended in 1 ml of 0.5% carboxymethyl cellulose (CMC) in distilled water to make the final concentration of 10, 30 and 100 mg/kg. The 0.5% CMC was given to the control group. The test compounds were orally given to the rats twice daily via intragastric gavage. All rats received 1 ml of either ECa 233 or 0.5% CMC, depending on the specified groups for 30 days.

### Behavioral study

After drug administration for 22 days, spatial learning and memory performance of all rats were assessed using the Morris water maze task. The diameter of a circular pool is 200 cm and the depth is 50 cm. The pool was filled with water up to 25-cm-depth. In the visible trial, the glass circular escape platform was shown 2 cm over the water surface and tested for four sessions only in the first day. The second day to the eighth day were the acquisition trial in which the visual cues were placed around three walls except the wall that the target quadrant was located. The glass circular escape platform was hidden 2 cm below the water surface. Rats were trained daily for seven days, consisting of four sessions per day. Rats were allowed to search for the escape platform for 120 seconds. The times to find the escape platform in both visible and acquisition trials were measured. Retention of the previously acquired memory was tested in the probe trial in which the glass platform was removed. Rats were allowed to swim, and the time spent in the target quadrant was measured to assess retention of the previously acquired memory.

### Quantification of bioactive constituents

#### Sample preparation

According to the study of Anukunwithaya in 2016, all plasma and brain tissues were prepared for protein precipitation by using methanol. Plasma was thawed at room temperature. Fifty μl of plasma from each rat was mixed with 200 μl of methanol and 10 ng of glycyrrhetic acids, which served as an internal standard. The mixture was centrifuged at 5000 × g for 10 min. Ten μl of the supernatant were collected and injected to the liquid chromatography tandem-mass spectrometry (LC-MS/MS) system. Fifty mg of each brain tissue were mixed with 200 μl of methanol and 10 ng of glycyrrhetic acids. The mixture was homogenized in an ice bath and centrifuged at 500 × g for 10 min. Then, the supernatants were collected and analyzed by the LC-MS/MS system.

#### Instrumentation

According to the study of Anukunwithaya in 2016^3^, the quantification of bioactive constituents was performed using the LC-MS/MS system. An Eksigent ekspert UHPLC 100 liquid chromatograph (Eksigent, Montreal, Canada) equipped with a QTRAP 6500 mass spectrometer and controlled by Analyst software, version 1.6 (AB Sciex, Pte. Ltd., Framingham, MA) was chosen for the LC-MS/MS system. The stationary phase was a Synergi Fusion-RP C18 column (Phenomenex, Inc.) with a 40 °C oven temperature. Methanol and 0.2% formic acid in water (pH 2.5) were used as the mobile phase in a gradient elution pattern at a flow rate of 0.5 ml/min as follows; 10% methanol for 0.5 min, increased to 90% methanol at 1.5 min for 2 min, then decreased to 10% methanol at 4.0 min for 4.5 min as the run time. Standards of madecassoside, asiaticoside and glycyrrhetinic acid were dissolved in methanol for tuning in the negative ion mode to obtain optimal MS conditions. The parameters for electrospray ionization were as follows; curtain gas, 25.0 units; collision gas, medium; ion spray voltage, −4500 V; temperature, 500 °C; ion source gas 1, 40.0 units; and ion source gas 2, 50.0 units. The MS conditions for the standard chemicals were tuned to establish the best conditions for the LC-MS/MS experiments. Using the optimized LC-MS/MS conditions, the retention times of madecassoside, asiaticoside, and glycyrrhetinic acid were 1.79, 1.82, and 2.12 min, respectively. Calibration curves of the two test compounds showed good linearity with correlation coefficients of R^2^ > 0.99 at 0.5–300 μg/l. Standard equations were used to calculate the amounts of active components in the biological samples. The lower limits of detection for madecassoside and asiaticoside were 0.1–0.5 μg/l, and the percentage recovery was over the range of 78–93% for all of compounds. The intra-day and inter-day precision and accuracy were within ±10%.

### Electrophysiology study

Extracellular long-term potentiation (LTP) recording was chosen for the synaptic plasticity assessment. After being anesthetized by isoflurane, the rat was decapitated quickly and the brain was removed rapidly and immediately. Hippocampal slices with 350 μm thickness were sectioned. Then the extracellular recording was performed under the light microscope with a 3 MΩ capillary recording electrode filled with aCSF (124 mM NaCl, 3 mM KCl, 1.25 mM NaH_2_PO_4_, 1.30 mM MgSO_4_-7H_2_0, 26 mM NaHCO_3_, 10 mM D-glucose and 2.4 mM CaCl_2_-2H_2_O). The recording electrode was positioned in the dendritic layer of area CA1 to record extracellular field excitatory post-synaptic potentials (fEPSPs). A tungsten stimulating electrode was placed 1000 μm away from the recording electrode but at the same dendritic layer to activate the Schaffer collateral commissural fibers. Recordings were made in response to a constant current single shock stimulation once every 30 sec. Stimulus intensities were adjusted to evoke fEPSPs of half maximal amplitude. The peak slope of the rising phase of the fEPSPs (1-ms duration) was calculated. Following 15 min of stable baseline activity recording, high frequency stimulation (2 trains of tetanic stimulation: 100 Hz of 100 pulses) was delivered at normal stimulus intensity to evoke LTP, and the activity was continually recorded for 180 min to observe LTP magnitude.

### Western blot analysis

After the rat brain removal, the hippocampal tissue was separated and incubated in the RIPA lysis buffer (50 mM Tris-HCl pH7.5, 10% of Triton X-100, 150 mM NaCl, 1 mM EDTA, 0.5% Na deoxycholate, 0.1% SDS, 1 mM PMSF), containing a protease inhibitor cocktail (Roche Molecular Biochemicals) for 30 min on ice. Tissue was homogenized by a sonicator (3 Hz frequency, 10 sec) for 2 times on ice. Tissue lysates were centrifuged at 14000 rpm for 15 min at 4 °C. The supernatant was collected and the protein concentration was measured by using Bradford assay (Bio-rad). Twenty micrograms of protein were mixed with 4x Laemmli sample buffer and denatured by heating at 95 °C for 5 min. Each protein sample was loaded onto 8% SDS-PAGE for NMDAR subunits (NR2A and NR2B) and tyrosine kinase B (TrkB); a receptor of BDNF, and loaded onto 12% SDS-PAGE for postsynaptic density-95 (PSD-95); a scaffolding protein of NMDAR and BDNF. The proteins were transferred to nitrocellulose membranes and then blocked by 3% non-fat dry milk in TBST (25 mM Tris–HCl (pH 7.5), 125 mM NaCl, 0.1% tween20) for 1 hr. After washing the membranes, specific primary antibodies were incubated with each membrane at 4 °C overnight (a mouse monoclonal anti-NR2A (sc-515148), a mouse monoclonal anti-NR2B (sc-365597), a mouse monoclonal anti-PSD95 (sc-32290) and a mouse monoclonal anti-β3 tubulin (sc-80016) (serves as internal control) were purchased from Santa Cruz biotechnology; a rabbit monoclonal anti-BDNF (ab108319) and a rabbit monoclonal anti-TrkB (ab187041) were purchased from Abcam). The membranes were washed and incubated with secondary antibodies for 1 hr (goat-anti mouse IgG (ab6789) and goat-anti rabbit IgG (ab6721) were purchased from Abcam). The antibody/protein complexes were enhanced by chemiluminescence substrate (Amersham ECL Prime Western blotting detection reagent; GE Healthcare) and detected by ImageQuant LAS4000 (GE Healthcare Life Sciences). Quantification of chemiluminescence intensity was determined using ImageJ software.

### Statistical analysis

The results were expressed as mean ± SEM. For the quantification of bioactive constituents, the data were evaluated by using one-way ANOVA followed by the LSD post hoc test. For the behavioral study, the data during the acquisition trial were evaluated by using one-way ANOVA with repeated measures followed by the LSD post hoc test. The data during the probe trial were evaluated by one-way ANOVA followed by the LSD post hoc test. For the electrophysiology study, the data were evaluated by using two-way ANOVA followed by the LSD post hoc test. For the quantification of the bioactive constituents and the western blot analysis, the data were evaluated by using one-way ANOVA followed by the LSD post hoc test. The differences between means of all statistical analyses were accepted as significant at the *p* value < 0.05.

## Supplementary information

Supplementary information of western blot analysis

## Data Availability

The datasets generated and analyzed during the current study are available from the corresponding author on a reasonable request.
