# Supplementary material for: Inverted U-shaped response of a standardized extract of Centella asiatica (ECa 233) on memory enhancement
Source: Sci Rep. 2019 Jun 10;9:8404. doi: 10.1038/s41598-019-44867-z (PMC6557898; doi:10.1038/s41598-019-44867-z)
Supplement: Supplementary file 1 — Supplementary information of western blot analysis [file 41598_2019_44867_MOESM1_ESM.pdf]

Inverted U-shaped response of a standardized extract of *Centella asiatica* (ECa 233) on memory enhancement

Yingrak Boondam[1], Phanit Songvut[2], Mayuree H. Tantisira[3], Sompol Tapechum[1], Kanokwan Tilokskulchai[1], Narawut Pakaprot[1]

[1]Department of Physiology, Faculty of Medicine Siriraj Hospital, Mahidol University, Bangkok, Thailand. [2]Department of Pharmacology and Physiology, Faculty of Pharmaceutical Sciences, Chulalongkorn University, Bangkok, Thailand. [3]Faculty of Pharmaceutical Sciences, Burapha University, Chonburi, Thailand.

Correspondence and requests for materials should be addressed to N.P. (email: narawut.pak@mahidol.ac.th

Supplementary Fig. S1. Western blotting of NR2A subunit in Figure 3A.

Supplementary Fig. S2. Western blotting of NR2B subunit in Figure 3B.

Supplementary Fig. S3. Western blotting of PSD-95 in Figure 4.

Supplementary Fig. S4. Western blotting of BDNF in Figure 5A.

Supplementary Fig. S5. Western blotting of TrkB in Figure 5B.

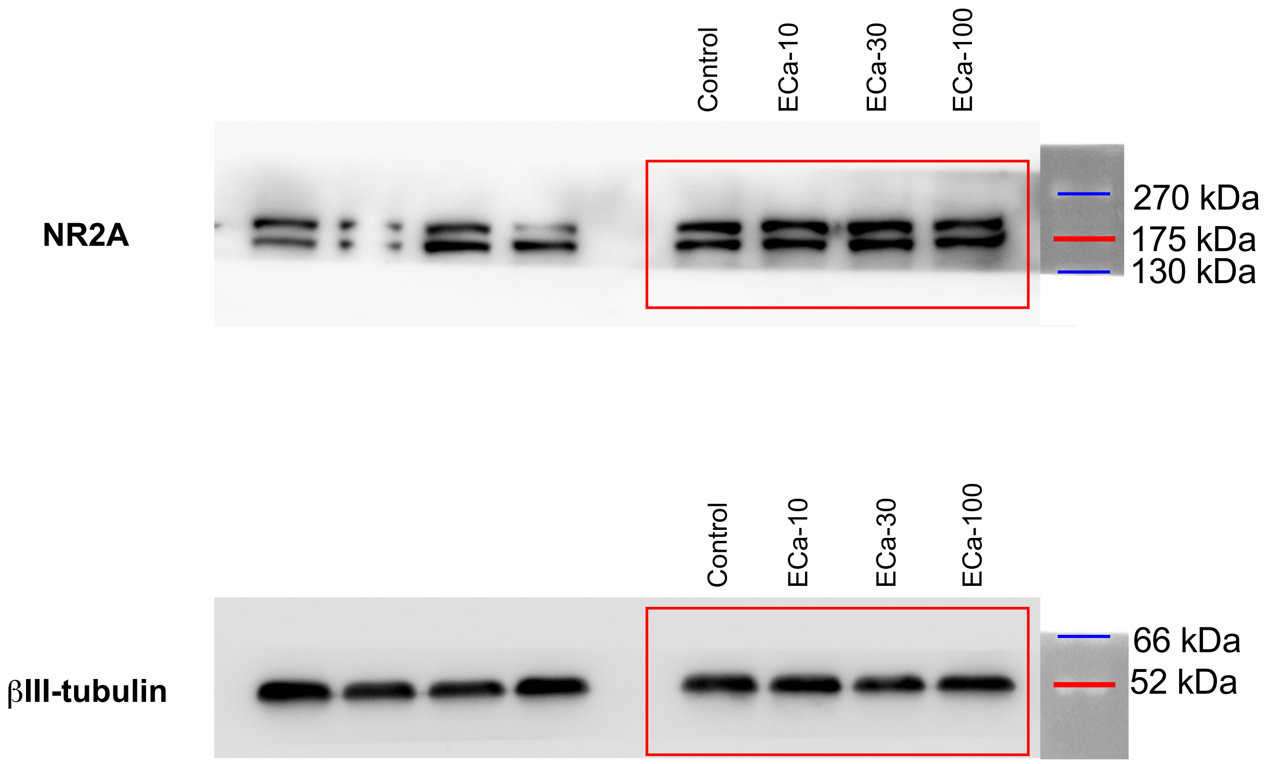

Supplementary Fig. S1.

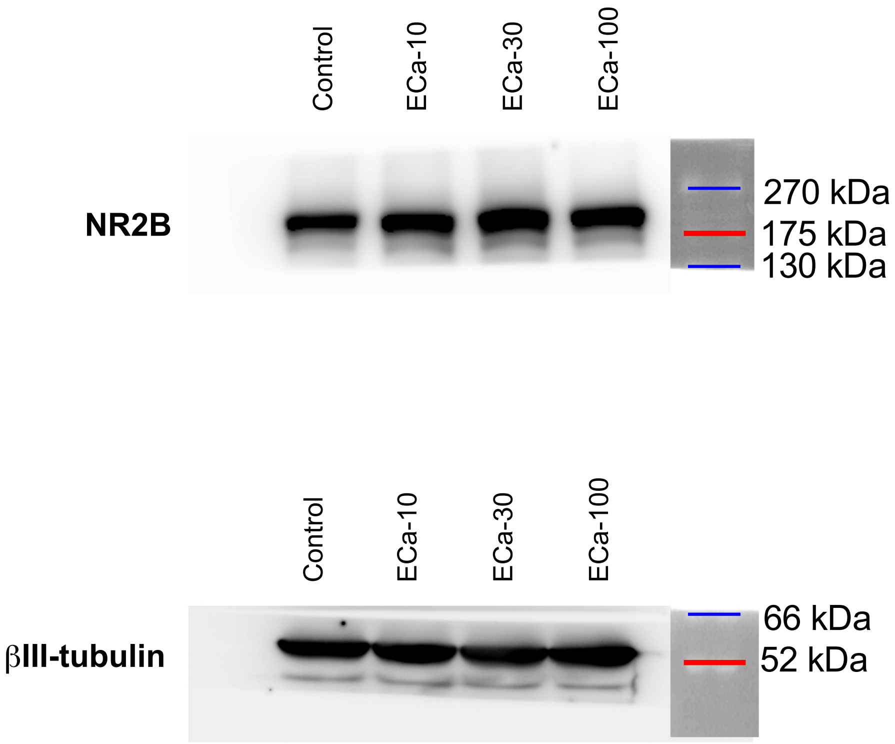

Supplementary Fig. S2.

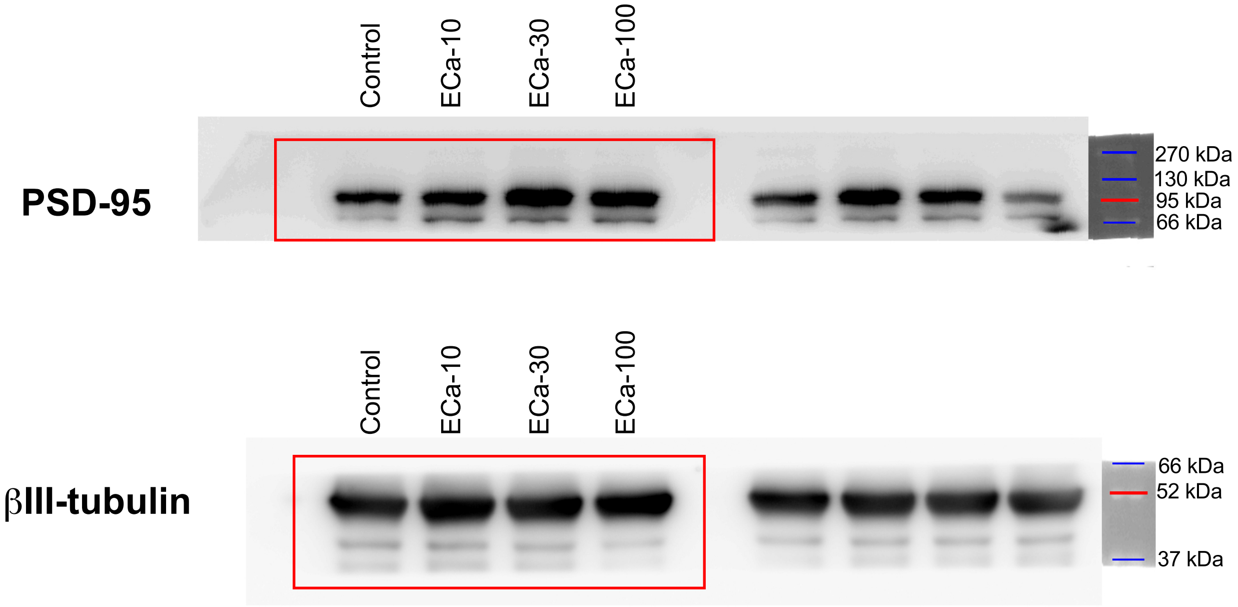

Supplementary Fig. S3.

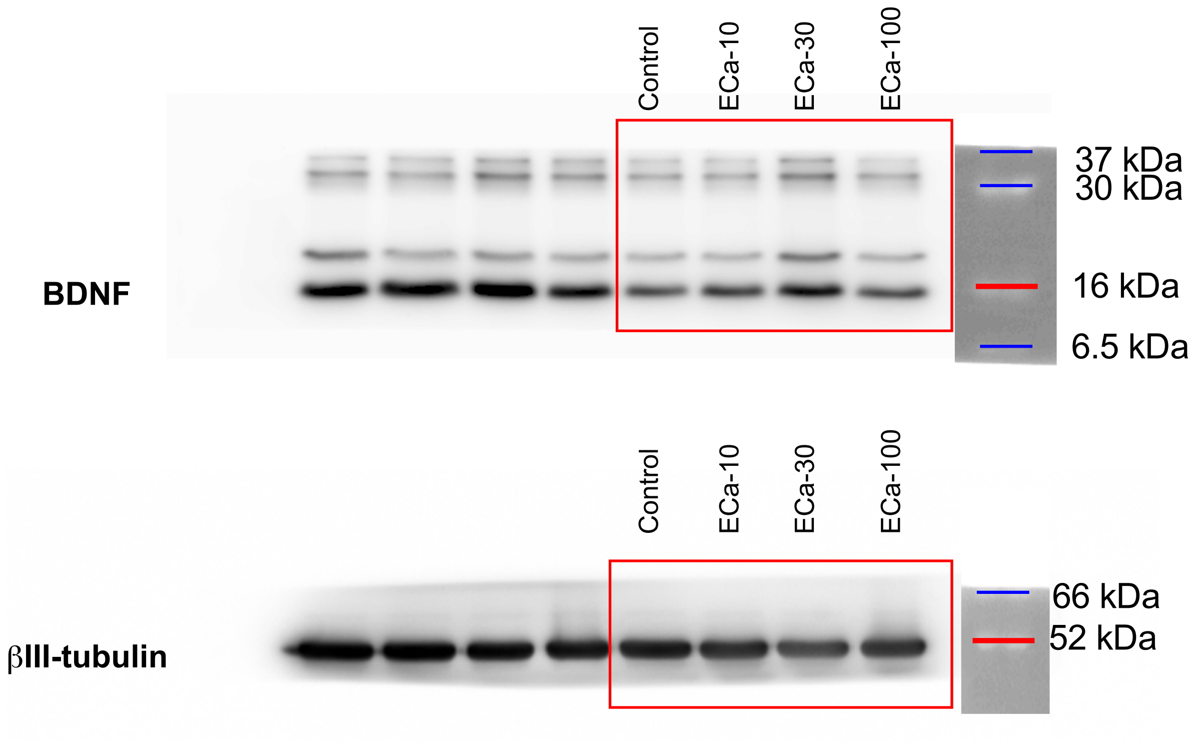

Supplementary Fig. S4.

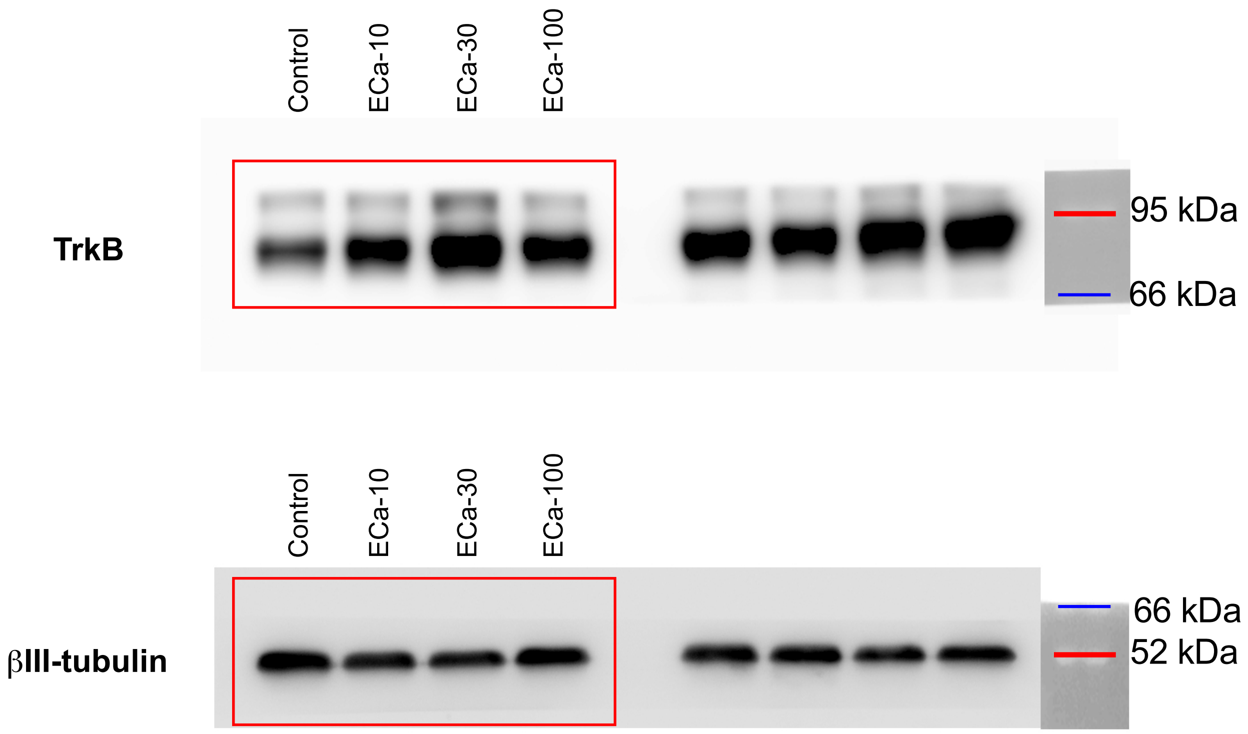

Supplementary Fig. S5.
